# Incidence of cervical disease associated to HPV in human immunodeficiency infected women under highly active antiretroviral therapy

**DOI:** 10.1186/1750-9378-4-9

**Published:** 2009-06-03

**Authors:** Martin Luther Koanga Mogtomo, Louise Carole Gouabe Malieugoue, Carolle Djiepgang, Michel Wankam, Andre Moune, Annie Ngono Ngane

**Affiliations:** 1 Laboratory of Viral Oncology, Department of Biochemistry, Faculty of Sciences, University of Douala, Cameroon; 2 Aids Care Unit and Gynaecology, Bonassama Hospital Douala, Cameroon; 3 Pathology Unit, Douala General Hospital, Cameroon

## Abstract

**Background:**

Women infected with human immunodeficiency virus (HIV) may be at higher risk of developing cervical cancer than non infected women. In a pilot study, we assessed the relationships among cervical cytology abnormalities associated to Human Papillomavirus (HPV), HIV infection and Highly Active Antiretroviral Therapy (HAART) on the development of Squamous Intraepithelial lesions (SILs). Out of the 70 HIV infected women from Douala -Cameroon (Central Africa) that we included in the study, half (35) were under HAART. After obtaining information related to their lifestyle and sexual behaviour, cervicovaginal samples for Pap smears and venous blood for CD4 count were collected and further divided into two groups based upon the presence or absence of cervical cytology abnormalities i.e. those with normal cervical cytology and those with low and high Squamous Intraepithelial lesions (LSIL, HSIL).

**Results:**

Assessment was done according to current antiretroviral regimens available nationwide and CD4 count. It was revealed that 44.3% of HIV-infected women had normal cytology. The overall prevalence of LSIL and HSIL associated to HPV in the studied groups was 24.3% (17/70) and 31.4% (22/70) respectively. Among the 22 HSIL-positive women, 63.6% (14/22) were not on antiretroviral therapy, while 36.4% (8/22) were under HAART. HIV infected women under HAART with positive HSIL, showed a median CD4+ T cell count of 253.7 +/- 31.7 higher than those without therapy (164.7 +/- 26.1). The incidence of HSIL related to HPV infection within the study group independently of HAART initiation was high.

**Conclusion:**

These results suggest the need for extension and expansion of the current study in order to evaluate the incidence of HPV infection and cervical cancer among HIV-infected and non HIV- infected women in Cameroon.

## Background

Genital infection by human papillomavirus (HPV) is one of the most common sexually transmitted infections, known to be the cause of cervical cancer [1-3]. Although HPV is known to be strongly associated with the development of cervical cancer, most HPV infections in young women are transient [4]. Women with persistent infection appear to have a higher risk of developing significant cervical cancer [2,5]. The burden of this infection on public health is compounded by the recognized causal relations between genital HPV infection and cervical dysplasia or cervical cancer [6,7]. Although factors that influence persistence of HPV are not yet well understood, several studies suggest that alterations in cell mediated immune responses play a major role in persistence of HPV. The higher rates of HPV infection, high-grade squamous intraepithelial lesion (HSIL), and cervical cancer among immunosuppressed individuals, specifically HIV-infected women, underscore the importance of control of immune response in HPV infection. Studies on adult women have consistently shown that the prevalence of HPV infection and HSIL are higher among HIV infected women and that these differences are exaggerated among women with lower CD4+ cell counts [8-10]. Several recent prospective studies have documented that the rate of persistence of HPV among HIV-infected women is higher than that among non HIV-infected women [11,12]. Specific types of HPV are associated with cervical cancer, but whether these high-risk types have natural histories that are different from those of other types not associated with cervical cancer is unknown [13,14]. The modification of the viral markers may be the crucial factor of disequilibrium in the interaction between virus and host: an increased replicating capacity of the virus versus a reduced control mechanism of the immune system. In this context it's evident that in HIV-infected women, both viral and host factors conspire, as these patients have an impaired immune system usually more exposed to HPV infection. Some studies have also considered the impact on cervical pathology on HIV disease care, mainly represented by the introduction of highly active antiretroviral therapy (HAART), which through the substantial recovery of immune function has significantly changed the scenario regarding HIV-related pathologies such as opportunistic infections and cancers [15,16]. The objectives of this study were to compare rates of cervical abnormalities related to HPV infection among HIV-infected women with and without HAART initiation and to examine immunological and behavioural risk factors associated with persistence of HPV.

## Subjects and methods

### Study population

A total of 70 HIV-infected women were enrolled in the study after informed consent and divided into two groups: 35 under HAART and 35 not initiated with HAART. Within eight (08) month, patients aged between 21–56 years, were recruited in a day care centre at Bonassama hospital in Douala for HIV therapy. All were included in a pilot study on clinical and behavioural characteristics associated with HIV infection. The study was carried out according to the guidelines for human experimental models in clinical research as stated by the ministry of public Health of Cameroon.

### Inclusion criteria

Enrolled women were single or married; non-pregnant; aged 21 years old and above and HIV infected, initiated or not with HAART. Patients were divided in two groups: group one consisted of women who were diagnosed HIV positive and were not yet eligible for antiretroviral therapy. Patients in this group were considered untreated only at the beginning of the study. The second group comprised HIV-infected women receiving HAART, who were enrolled on the basis of a three months' minimum length of treatment. Patients in this group had different therapeutic protocols spread over different periods of treatment. From the list of patients eligible for the study, subjects were randomly chosen in a systematic manner. The objective of the study was explained to patients and verbal consent was sought from each of them.

### Collection of Specimens

At the screening visit, venous blood was collected for CD4 analysis, and pap smears performed for early detection of cervical carcinoma as previously described [17]. Subjects underwent a general physical examination and completed a short standardized interview, including questions pertaining to medical history, sexual behaviour, history of STIs, age of first sexual intercourse, pregnancy, parity and abortion.

### Histological analysis

Pap smears were interpreted and classified according to the Bethesda System as negative, atypical squamous cells of unknown significance, LSIL, HSIL, or invasive cervical cancer [18,19]. Conventional Pap smears were used and slides were read by a pathologist. Blood samples were obtained for flow cytometry CD4+ T cell counts using AIDS Clinical Trials Group standardized flow-cytometry protocols as described elsewhere [20].

### Statistical analysis

Data obtained were verified for consistency, coded, and computerized. Throughout the text, values are given as mean ± s.d. Percentages were calculated from the overall number of cases. Raw data were compared using Fisher's exact-test (StatXact 2.05 software). Appropriate probabilities were calculated [21] and size variation with Mann-Whitney rank sum test as the normality test failed (Sigma Stat 2.03 software).

## Results

### Subjects characteristics

Seventy HIV infected women were randomly recruited using systemic random sampling from the outpatients at the day care centre of Bonassama hospital in Douala. Table 1 summarizes the socio-demographic information as well as information on reproductive and sexual characteristics of the study groups. Sampling was done according to: age with a mean age of 35.5 ± 1.9 years and 35.1 ± 1.3 years; age of first vaginal sex with a mean age of 16.9 ± 5 years and 17.2 ± 0.5 years; number of pregnancies with a mean number of 3.7 ± 0.5 and 3.9 ± 0.4; parity with a mean number of 2.6 ± 0.5 and 2.6 ± 0.4; abortion with a mean number of 1.2 ± 0.2 respectively for women under HAART and women not initiated with HAART. Significant differences were observed between these groups at baseline in terms of ages of patients: when comparing the mean age of patients above 40 years (52.3 ± 2.3% (9) vs. 44.9 ± 1.2% (10), P = 0.007) and when comparing the mean parity of more than 4 (9.8 ± 0.7% (4) vs. 5.9 ± 0.5% (8), P = 0.006) for treated and untreated group respectively. The significant difference observed between treated and untreated groups in terms of mean age and parity is probably due to the number patients assigned to each parameter.

**Table 1:** Selected baseline characteristics of HIV infected women as study subjects.

	**Under HAART**			**No HAART**			
			
**Groups**	n	%	Mean ± SE^a^	n	%	Mean ± SE	p value
A. Age (years)							
<30	12	34.3	26.4 ± 0.7	10	28.6	26.4 ± 0.9	0.988 ns
30 – 40	14	40.0	32.4 ± 0.8	15	42.8	34.3 ± 0.9	0.115 ns
>40	9	25.7	52.3 ± 2.3	10	28.6	44.9 ± 1.2	0.007 *
Total	35	-	35.5 ± 1.9	35	-	35.1 ± 1.3	0.872 ns
B. Age at first vaginal sex (years)							
≤ 16	18	51.4	14.6 ± 0.3	15	42.9	14.5 ± 0.3	0.874 ns
>16	17	48.6	19.3 ± 0.7	20	57.1	19.3 ± 0.4	0.956 ns
Total	35	-	16.9 ± 0.5	35	-	17.2 ± 0.5	0.643 ns
C. Pregnancy							
0	2	5.7	0.0 ± 0.0	0	0.0	0.0 ± 0.0	-
1 – 4	23	65.7	2.3 ± 0.3	22	62.9	2.3 ± 0.2	0.971 ns
5 – 9	7	20.0	6.1 ± 0.6	11	31.4	5.9 ± 0.4	0.750 ns
>10	3	8.6	11.0 ± 0.6	2	5.71	10.5 ± 0.5	0.800 ns#
Total	35	-	3.7 ± 0.5	35	-	3.9 ± 0.4	0.737 ns
D. Parity							
0	7	20.0	0.0 ± 0.0	5	14.3	0.0 ± 0.0	-
1 – 4	24	68.6	2.1 ± 0.3	22	62.8	2.0 ± 0.2	0.711 ns
>4	4	11.4	9.8 ± 0.7	8	22.8	5.9 ± 0.5	0.006 *
Total	35	-	2.6 ± 0.5	35	-	2.6 ± 0.4	0.964 ns
E. Abortion							
0	13	37.1	0.0 ± 0.0	12	34.3	0.0 ± 0.0	-
1 – 2	15	42.9	1.2 ± 0.1	17	48.6	1.4 ± 0.1	0.353 ns
>2	7	20.0	3.4 ± 0.2	6	17.1	3.3 ± 0.3	0.628 ns
Total	35	-	1.2 ± 0.2	35	-	1.2 ± 0.2	0.925 ns

a. Means were compared one after the other using the Student t-test when normality and equal variance conditions passed or the Mann-Whitney rank sum test when conditions failed.

Ns: no significant difference; * significant difference; #: Mann-Whitney rank sum test

### HAART and incidence of cervical abnormalities

Table 2 presents histological analyses of studied samples. The Pap smear test results revealed the following within the study groups: those with normal cervical cytology (44.3%) and those with abnormal Pap smear; LSIL (24.3%), and HSIL (31.4%) respectively. The cervical abnormalities differences were statistically significant for the two study groups when comparing the total frequency abnormalities (48.6% vs. 62.9%, P = 0.034) for treated and untreated groups respectively. Table 3 represents the frequency of distribution of cervical cytology results in the treated and untreated groups according to the duration of therapy. Cervical abnormalities are high in the absence of therapy: 47.1% and 63.6% for LSIL and HSIL respectively. The difference in the distribution of the cytological picture among LSIL and HSIL cases was statistically none significant (P > 0.05). Table 4 represents the Incidence of cervical cytology results in the HAART treatment group according to the duration of therapy. Normal cervical cytology increase with the duration of therapy: 5.7% to 34.3% within the period of treatment 1–5 month and more than 10 month respectively. Within the same period, HSIL decreased from 14.3% to 2.8% and was probably due to the number of patients within the more than 10 months' period of therapy. The difference in the incidence of cervical abnormalities was statistically non significant (P = 0.069). Table 5 represents CD4+ T cell count distribution within the study groups according to cervical abnormalities. The mean of CD4+ T cell count decreases with degree of cervical abnormalities in both groups: 289 ± 47.0, 253 ± 69.1 and 173.4 ± 42.4 T cells in patients under HAART while those with no HAART initiation have 244.5 ± 42.8, 218.4 ± 64.1 and 59.9 ± 14.5 for normal, LSIL and HSIL respectively. Table 6 examines some of the risk factors that might contribute to HPV infection. The risk factors investigated were grouped according to the following parameters of personal history: age of first vaginal sex, pregnancy and abortion. The results showed that patients with cervical abnormalities were mainly ≥ 19 years old at first vaginal sex intercourse with high percentage: 23.5% and 20.2% for LSIL and HSIL respectively. There was no statistical difference for this factor when compared with Mann-Whitney test. Reproductive history of the studied groups showed a statistically non significant difference between the LSIL and HSIL positive patients neither for pregnancy nor for abortion. In patients with the highest percentage for pregnancies, i.e. ≤ 10 pregnancies we noticed 76.4% and 45.5% occurrence for LSIL and HSIL respectively while for abortion, the cytology results findings showed 70.6% and 36.4% for LSIL and HSIL respectively in patients with 1 or more than 2 abortions. Table 7 represents distribution of STI risk factors that might contribute to HPV infection. The history of STIs risk factors shows that: *Bacillus vaginalis, Chlamydia sp *and *Treponema Pallidum *are main microbial infectious agents. *B. vaginalis *is the leading infection with 70.6% and 54.5% for LSIL and HSIL respectively while *Chlamydia *is the second with 17.6% and 22.7% and *T. Pallidum *which count for 5.9% and 13.6% respectively for LSIL and HSIL positives patients. No significant statistical difference was found in correlation with cervical abnormalities.

**Table 2:** Frequency of cervical abnormalities in HIV infected women according to cytological findings

	**Under HAART(35)**		**No HAART (35)**		**Total (70)**		**p-value**
				
**Groups**	n	%	n	%	n	%	
**Normal**	18	51.4	13	37.1	31	44.3	
**LSIL**^a^	9	25.7	8	22.9	17	24.3	
**HSIL**^b^	8	22.9	14	40.0	22	31.4	

**Total abnormalities**	**17**	**48.6**	**22**	**62.9**	39	55.7	0.034*

a. Low squamous intra epithelial lesions b. High squamous intra epithelial lesions

* Significant difference

**Table 3:** Frequency of cervical abnormalities in HIV infected women according to duration of medication (HAART)

**Duration**	**Cervical abnormality**^a^				
		
(month)	LSIL		HSIL		
			
	n	%	n	%	Exact p-value^b^
0	08	47.1	14	63.6	0.1308 ns
1–5	03	17.7	05	22.7	0.6193 ns
6–10	02	11.8	02	09.1	1.000 ns
> 10	04	23.5	01	04.5	0.2063 ns

Total	17	-	22	-	0.3652 ns

a. Combined data for treated and untreated patients

b. Global comparison of raw data: Pearson's chi-square exact probability: P = 0.3725)

**Table 4:** Cervical abnormalities incidence in HIV infected women under HAART therapy according to and duration of medication

**Duration**	**Normal**		**LSIL**		**HSIL**	
			
(month)	n	%	n	%	n	%
0	-	-	-	-	-	-
1–5	2	5.7	3	8.6	5	14.3
6–10	4	11.4	2	5.7	2	5.7
> 10	12	34.3	4	11.4	1	2.8

Total	18	51.4	9	25.7	8	22.8

a. exact probability using Pearson's Chi-square test for independence procedure: P = 0.069

**Table 5:** CD4+ T cell count distribution in HIV infected women according to cervical abnormalities

	**Under HAART**			**No HAART**		
		
**Groups**	n	%	Mean ± SE	n	%	Mean ± SE
**Normal**	18	51.4	289.4 ± 47.0	13	37.1	244.5 ± 42.8
**LSIL**	9	25.7	253.6 ± 69.1	8	22.9	218.4 ± 64.1
**HSIL**	8	22.9	173.4 ± 42.4	14	40.0	59.9 ± 14.5

Total	35	-	253.7 ± 31.7	35	-	164.7 ± 26.1

**Table 6:** Incidence of risk factors on cervical disease in HIV infected women under treatment (HAART) and those without treatment or HAART initiation

	**LSIL**			**HSIL**			Mann-Withney test
			
**Groups**	n	%	Mean ± SE	n	%	Mean ± SE	
**Age at first vaginal sex (years)**							
<15	03	17.6	13.7 ± 0.3	06	27.3	13.2 ± 0.3	0.381 ns
15 – 18	10	58.8	16.5 ± 0.5	11	50.0	16.4 ± 0.3	0.860 ns
≥ 19	04	23.5	19.8 ± 0.5	05	22.7	20.2 ± 0.7	0.905 ns

Total	17	-	16.8 ± 0.5	22	-	16.4 ± 0.6	0.571 ns

**Pregnancy**							
≤ 4	13	76.4	2.4 ± 0.3	10	45.5	2.8 ± 0.3	0.420 ns
5–9	02	11.8	5.5 ± 0.5	10	45.5	1.4 ± 0.4	0.590 ns
≥ 10	02	11.8	11.5 ± 0.5	02	09.0	0.7 ± 0.5	0.333 ns

Total	17	-	3.8 ± 0.8	22	-	5.1 ± 0.6	0.068 ns

**Abortion**							
0	03	17.6	0.0 ± 0.0	06	27.3	0.0 ± 0.0	-
1–2	12	70.6	1.3 ± 0.3	08	36.4	1.4 ± 0.2	0.669 ns
> 2	02	11.7	3.0 ± 0.0	08	36.4	3.6 ± 0.3	0.400 ns

Total	17	-	1.24 ± 0.2	22	-	1.8 ± 0.3	0.372 ns

**Table 7:** Incidence of History of STD on cervical disease in HIV infected untreated and treated women (exact probability using Pearson's Chi-square test for independence procedure: P = 0.8224)

	**LSIL**		**HSIL**	
		
**Groups**	n	%	n	%
*B. vaginalis*	12	70.6	12	54.5
*Chlamydia*	03	17.6	05	22.7
*T. Pallidum*	01	05.9	03	13.6
Other	01	05.9	02	09.1

Total	17	-	22	-

## Discussion

This study provides data on the risk of cervical cancer among HIV positive women less than 60 years old in the day care centre at Bonassama hospital Douala -Central Africa. The extent to which HIV increases the risk for cervical cancer is especially important in Cameroon referring to the epidemiology of the HIV especially among women. In our study, HIV positive women are at high risk of invasive cervical cancer. This could be due to the competing risk of mortality from other conditions associated with HIV/AIDS, particularly in a setting where antiretroviral therapy was not available at the time and for all patients. Our findings that HIV infected women were at a significantly higher risk of LSIL and HSIL confirm the results of studies carried out in both developed and other developing countries [7,17,22-24]. The relative prevalence of SILs or cytological abnormalities among HIV positive women is higher than that ever been reported by any other study in Africa. Hawes *et al*. reported a cytological abnormality prevalence of 37% among women with HIV-1 infection attending an outpatients infectious disease clinic in Senegal [17], Yamada 27.1% in urban patients in Kenya [25], Moukassa 15.36% among urban dwellers in Congo [26].

A potential limitation of this current study is that cytological abnormalities were not histologically confirmed. However, it has been shown that the Pap smear sensitivity and specificity are similar among HIV negative and HIV positive women [27]. We found an association between HAART and the stage of cervical pre-cancer. Our results are in agreement with others that have shown an association between HIV, HAART and cervical abnormalities [[15,16], and [28]]. An additional limitation of our study is that complete information regarding the immunological status of patients has not been documented for all the groups. Similar of different studies by the same author [[9,15,16,20,25], and [29]] have reported that the severity of HIV-related immunodepression is associated with increased incidence of HPV infection and SILs. Data obtained from the current study further confirm these findings reported by other investigators of our geographic area [17,22-26]. Several studies suggested that markers of advanced HIV disease such as CD4+ T cell counts or HIV-RNA plasmatic levels are associated with increased risk of HPV infection and SIL [8,17,28,29]. However some documentations and data have revealed that the relationship between HIV and HPV is more complex. Human immunodeficiency virus infection is associated with an increased transcriptional activity of early HPV genes. Studies on adult women have shown that HPV infection with both high-risk and low-risk HPV types is more likely to persist among HIV-infected women [8,12]. Because they have more years of sexual activity, adult women may reflect a group of women further along in their history of HIV and HPV infection. Our data further support the important role played by CD4+ T cells [28,29] in the control of HPV infection: the lower the CD4+ T cell count, the more likely that HPV infection will persist. Persistent infection in turn may increase the risk for the development and persistence of squamous intraepithelial lesions. In fact, women whose immunosuppression is related to infection with human immunodeficiency virus are at increased risk of infection with multiple types of HPV [28]. In developed countries it's recommended for HIV positive women to have two cervical cytological assessments within the first year after HIV diagnosis and annually thereafter, referred for colposcopy for any smear showing an ASCUS (atypical squamous cells of undetermined significance) or more severe lesions [30]. These guidelines are not feasible in settings with limited or poor resources. The high prevalence and risk of cervical abnormalities documented in our research projet underscores the importance of developing screening and management guidelines for HIV positive women. As antiretroviral therapy becomes increasingly available in Cameroon, the life expectancy of HIV positive women will increase as well. It's therefore important to conserve this invaluable benefit and avoid its upset by an increased risk in the development of cervical cancer. A cervical screening program is of critical importance as it informs local researchers on the natural history of cervical abnormalities, which might be recommended for HIV positive women. The STIs history of studied subjects revealed the presence of reproductive tract infection in patients with cervical cytology abnormality. This high rate of infection is attributed to the promiscuous sexual behaviour of the sampled women. Other studies suggest lack of hygiene, crowded or unsanitary living conditions, stress, and the presence of other sexually transmitted infections as factors that spread HPV infection and, eventually cervical cancer [30]. In the current study, history of STI infections was a non significant risk predictor in distinguishing LSIL to HSIL positive cases.

## Conclusion

HPV infection increased in accordance with lower CD4+ T cell counts and higher HIV RNA levels. High-grade lesions were strongly associated with high-risk HPV infection and low CD4+ T cell counts. Immunodeficiency as a result of HIV infection appears to be important for malignant progression within the cervix. Nationwide prevention of HIV infection and cervical cancer screening are necessary for the health of women in Cameroon. The increased public health burden enhanced by HPV is an important and gender-specific aspect of HIV infection. Guidelines on proper screening for cervical abnormalities in HIV positive women are urgently needed in Cameroon.

## Competing interests

The authors declare that they have no competing interests.

## Authors' contributions

KMML conceived the study, participated in its design and statistical analysis, coordinated and drafted the manuscript. GMLC participated in CD4 analysis using the flow cytometry, data collection and helped to draft the manuscript. DC participated in flow cytometry analysis, data collection and helped to draft the manuscript. WM participated in study design, clinical examination and recruitment of women at the study site and coordination. MA carried out cytology analysis. NNA participated in the study design, statistical analysis and helped to draft and review the manuscript.
